# A hybrid *in situ* and on-screen survey to monitor gully erosion across the European Union

**DOI:** 10.1038/s41597-025-05074-w

**Published:** 2025-05-07

**Authors:** Pasquale Borrelli, Francis Matthews, Christine Alewell, Konstantinos Kaffas, Jean Poesen, Philipp Saggau, Remus Prăvălie, Matthias Vanmaercke, Panos Panagos

**Affiliations:** 1 Department of Science, Roma Tre University, Rome, Italy; 2 Department of Environmental Sciences, Environmental Geosciences, University of Basel, Basel, Switzerland; 3 Department of Earth and Environmental Sciences, KU Leuven, Leuven, Belgium; 4 Maria-Curie Sklodowska University, Institute of Earth and Environmental Sciences, Lublin, Poland; 5 University of Bucharest, Faculty of Geography, Bucharest, Romania; 6 University of Bucharest, Research Institute of the University of Bucharest (ICUB), Bucharest, Romania; 7 Academy of Romanian Scientists, Bucharest, Romania; 8 European Commission, Joint Research Centre (JRC), Ispra, Italy

**Keywords:** Environmental impact, Geomorphology

## Abstract

After the successful mapping of gully erosion channels in the 2018 Eurostat Land Use/Cover Area Frame (topsoil) statistical survey (LUCAS, n = 24,759 locations), the methodology was further expanded across the full LUCAS 2022 survey (n = 399,591 locations). This expert-based assessment identifies the presence or absence of gully erosion forms at each LUCAS location. Its goal is to improve understanding of gully erosion geography in the EU and develop forecasting methods to support soil health indicators proposed by the new Directive on Soil Monitoring and Resilience (COM(2023)416) and Common Agricultural Policy monitoring. Here, we present the findings of our analysis which led to the development and validation of the LUCAS Gully Erosion Model (GE-LUCAS v1.1), a pan-European inventory of gully erosion channels comprising 3,116 locations (~0.8% of all monitored locations) affected by gully erosion throughout the European Union. We further present gully erosion patterns and provide insights on how GE-LUCAS v1.1 inventory can be used to estimate the probability of gully occurrence in areas beyond the monitored locations.

## Background & Summary

The Eurostat Land Use/Cover Area frame statistical Survey, in short LUCAS, is a mainly *in situ* survey program running every three years since 2006 (n = 6) across the European Union (EU). It is designed to produce harmonised statistics for all 27 EU Member. Between 2006 and 2018, LUCAS surveyed a total of 1,351,293 points at 651,780 unique locations, collecting data on 106 variables. The latest 2022 survey campaign lasted five months and involved several actors, including more than 500 surveyors. Out of the 399,591 locations surveyed in 2022, 49.8% (n = 199,043) were surveyed *in situ*, while the remaining 50.2% (200,548 locations) were surveyed through the interpretation of orthophotos. The survey consists of five modules: LUCAS Core (2006, 2009, 2012, 2015), Soil Module (2009, 2015, 2018 and 2022), Grassland Module (2018 and 2022), Copernicus Module (2018 and 2022), and Landscape Feature Module (2022). The LUCAS 2022 survey increased the data collection, which raised to 265 different variables at a total of 399,591 locations. In addition, for each LUCAS locations, the surveyors walked along a 250-meter straight line transect (east direction), recording each observed land cover transition according to the LUCAS protocol^1^.

Particularly relevant for the EU Soil Strategy, which aims to achieve healthy soils by 2050 and concrete actions to be implemented by 2030, was the introduction of a Soil Module starting from the LUCAS 2009 survey. Generally referred to as ‘LUCAS Soil’ or ‘LUCAS Topsoil’, this survey comprised a soil sampling procedure focused on the topsoil (0–20 cm depth) of approximately 10% of all LUCAS locations^2^. Its main objective is to record spatiotemporal changes in soil physical-chemical properties across the EU. In 2018, the Soil Module was further expanded introducing variables focusing on soil biodiversity (n = 885 locations), bulk density (n = 6,140 locations), and gully erosion (n = 24,759 locations)^3^.

Gully erosion channels usually form on hillsides, foothills, and to a lesser extent, on valley bottoms when soil is incised by concentrated overland flow^4^. They primarily form or expand during heavy rainfall events, when overland flow occurs by infiltration-excess^5^. Gullies, being highly dynamic landforms, frequently result in a diverse range of on-site and off-site effects, including the formation of Badlands^5,6^. The LUCAS 2022 survey (available for free download at https://ec.europa.eu/eurostat/web/lucas/database/2022) extended the visual detection of gully erosion channels to all 399,591 locations of the monitoring grid across the EU. The survey focused on gully channels because, among all soil erosion processes induced by the combined action of rainfall and runoff —splash, sheet (or interrill), rill, and gully erosion^7^— gullies stand out as the most severe erosion forms but, at the same time, as a hitherto poorly understood threat^8,9^. The mechanisms of gully erosion are complex due to their threshold-dependent nature, as well as the interactions of hydraulic (sub-)surface and mass wasting processes. As a result, they remain difficult to replicate in erosion prediction models^9–11^. On the other hand, gullies, despite being polymorphic and highly dynamic land degradation forms^12^, exhibit distinct morphological features that make them easily detectable^3,6^. A condition that makes gully erosion channels a suitable target for both *in situ* and on-screen remote mapping^13^ and, therefore, a suitably compatible with match for the LUCAS survey.

**Fig. 1:**
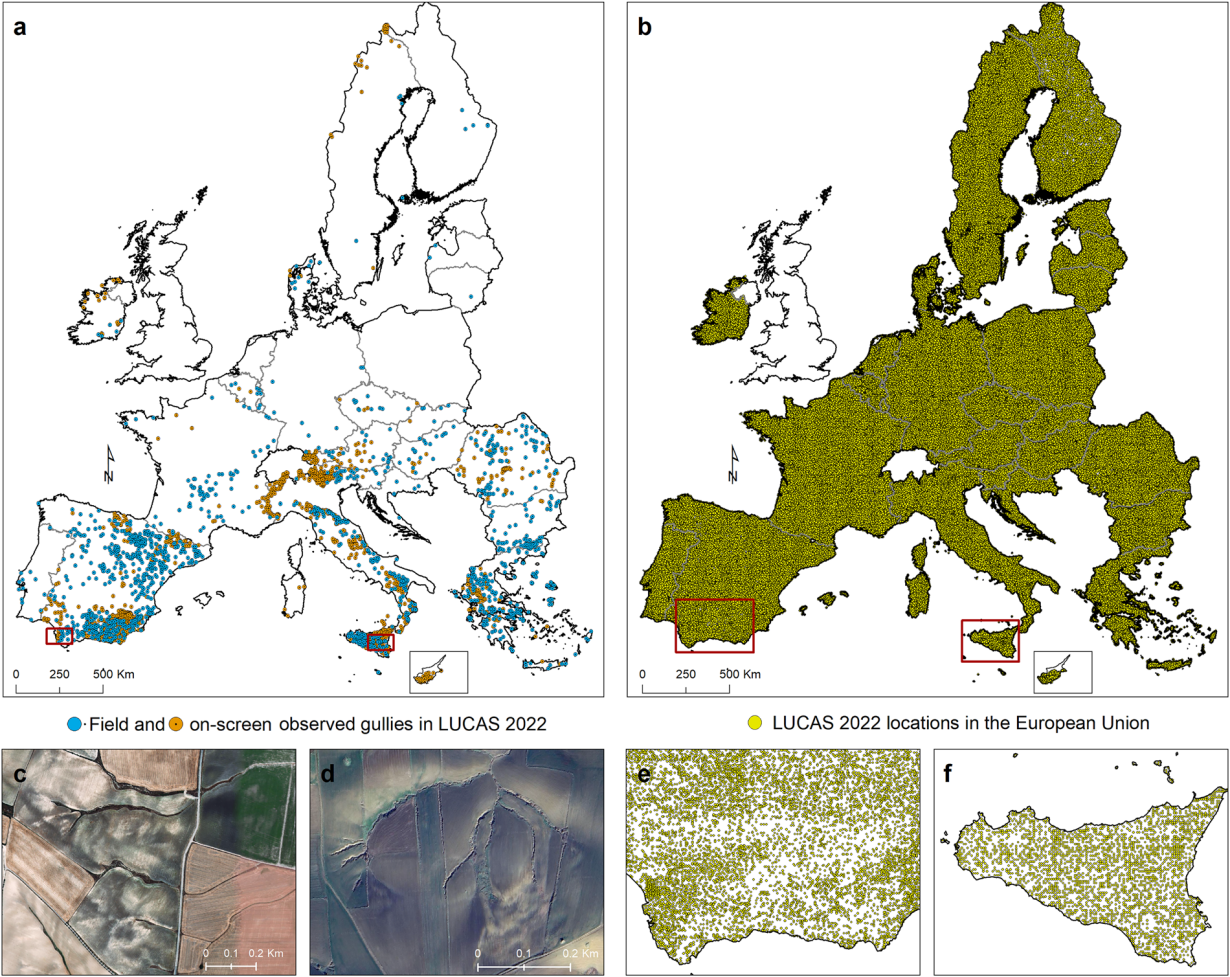
LUCAS Topsoil survey 2022. (**a**) Locations where gully erosion channels were observed in situ (orange colour) or via on-screen interpretation (blue colour). (**b**) Spatial distribution of 399,591 LUCAS Topsoil survey locations visited in 2022 in the EU. Locations (red areas in panel **a**) exhibiting prominent signs of gully erosion channels in Spain (Andalusia) (**c**), and Italy (Sicily) (**d**). Detailed view on the LUCAS 2022 locations in Spain (Andalusia) (**e**) and Italy (Sicily) (**f**) based on the red boxes in panel **b**.

Borrelli *et al*.^3^ described the results of 2018 achieved through the gully erosion monitoring in the pilot testing at 24,759 LUCAS locations. Gully erosion channels were detected in ~1% (n = 211 locations) of the sites The results of the cross-checking procedure showed low commission (false positives, 2.5%) and omission errors (false negatives, 5.6%), which were deemed insufficient to compromise the ability of the survey to give a first geographical overview of gully erosion. During the LUCAS 2022 feature detection survey, gully erosion channels were observed at 3,116 out of the 399,591 LUCAS monitored locations, which corresponds to ~0.8% of the total (Fig. 1a). A figure comparable to what Borrelli *et al*.^3^ found, for the LUCAS 2018 topsoil locations. Out of the 3,116 locations, 1,649 (~53%) were detected via on-screen imagery interpretation, while the remaining ~47% were mapped by *in situ* observations. As highlighted by Borrelli *et al*.^3^, and inferable from Fig. 1a, the locations with gully channels indicate a distinction of the EU into three large regions: (i) a northernmost region with few observations, (ii) a central eastern region with a moderate number of observations, and (iii) a Mediterranean region with predominance of medium to high numbers of observations. The largest number of detected gully erosion channels was found in Spain (n = 1,148 locations, 37%), with the highest concentration in the Andalusian region (n = 441 locations, 14%). Spain, Italy (34%), and to a lesser extent Greece (9%) and Romania (5%), are the countries with the highest incidence of mapped gullies (together 85%). Regarding land use/cover (Table 1), shrubland shows the higher gully occurrence (~2.3% of LUCAS samples), followed by bare land (~2%), grassland (~0.86%), and woodland (0.66%). Cropland shows the lowest gully occurrence rate with 0.38%. These findings are consistent with the relative presence of gullies in shrubland (~3.4%), bare land (~1.3%), forest (~0.8%), cropland (~0.7%), and grassland (~0.6%) observed in the 2018 LUCAS topsoil campaign. A closer look to the land use/cover categories reveals that gullies have a high occurrence in olive orchards, accounting for ~25% of all LUCAS gully observations. Andalusia (Spain) is the region with the highest incidence of gully observations in olive groves because of the combination of factors: sloping hillsides, very erodible soils (clayey texture), high erosivity rains and mostly bare and cultivated topsoils (Hayas *et al*. 2017) where land management often aims at keeping orchards free of understorey vegetation to reduce soil moisture competition. Concerning the biogeographical regions (Table 2), the Mediterranean is by far the region with the highest frequency of gully observations (~2.5% of LUCAS samples observed in this region), followed by the Alpine region (~1.6%). Results also indicate that gullies are more likely to occur in specific soil textures (Table 3), i.e., clay (~9.7% of LUCAS samples observed in this class, n = 70), silty clay (~8.2%, n = 94), clay loam (~1.9%, n = 777), and to a lesser extent silty clay loam (~1.1%, n = 180), sandy clay loam (~1%, n = 34), and loam (0.98%, n = 1,595) (USDA classification after Ballabio *et al*.^14^). These preliminary spatial analysis results highlight the geographic distribution of gully erosion, revealing key patterns across Europe. The higher occurrence of gullies in Mediterranean regions, especially in Spain and Italy, underscores the significant role of local factors such as soil texture, land use, and climate.

**Table 1:** Distribution of LUCAS 2022 locations with gullies by land cover category.

Soil textures	Locations with gullies	LUCAS locations [n]	Gully occurrence [%]
Artificial	53	19,459	0.27
Cropland	372	98,078	0.38
Woodland	734	110,477	0.66
Shrubland	701	30,159	2.32
Grassland	867	100,375	0.86
Bare land	328	16,133	2.03
Wetlands	61	24,910	0.24
Total	3116	399,591	0.78

**Table 2:** Distribution of LUCAS 2022 sites with gullies by biogeographical region.

Bio-geographical region	Locations with gullies	LUCAS locations [n]	Gully occurrence [%]
Alpine	561	35,938	1.56
Atlantic	93	63,070	0.15
Black Sea	3	1,141	0.26
Boreal	14	49,687	0.03
Continental	401	154,195	0.26
Mediterranean	1,998	79,247	2.52
Pannonian	29	12,018	0.24
Steppic	17	4,295	0.40
Total	3,116	399,591	0.78

**Table 3:** The number of locations with gullies aggregated by USDA soil texture class.

Soil texture class	Locations with gullies	LUCAS locations [n]	Gully occurrence [%]
Clay	70	720	9.72
Silty clay	94	1,141	8.24
Silty clay Loam	180	1,6167	1.11
Sandy clay	0	0	0.0
Sandy clay Loam	34	3,284	1.04
Clay loam	777	41,966	1.85
Silt	0	0	0.0
Silt loam	163	54,668	0.30
Loam	1,595	162,732	0.98
Sand	2	3,745	0.05
Loamy sand	4	16,673	0.02
Sandy loam	133	90,949	0.15
Not specified	64	7,546	0.85
Total	3,116	399,591	0.78

**Fig. 2:**
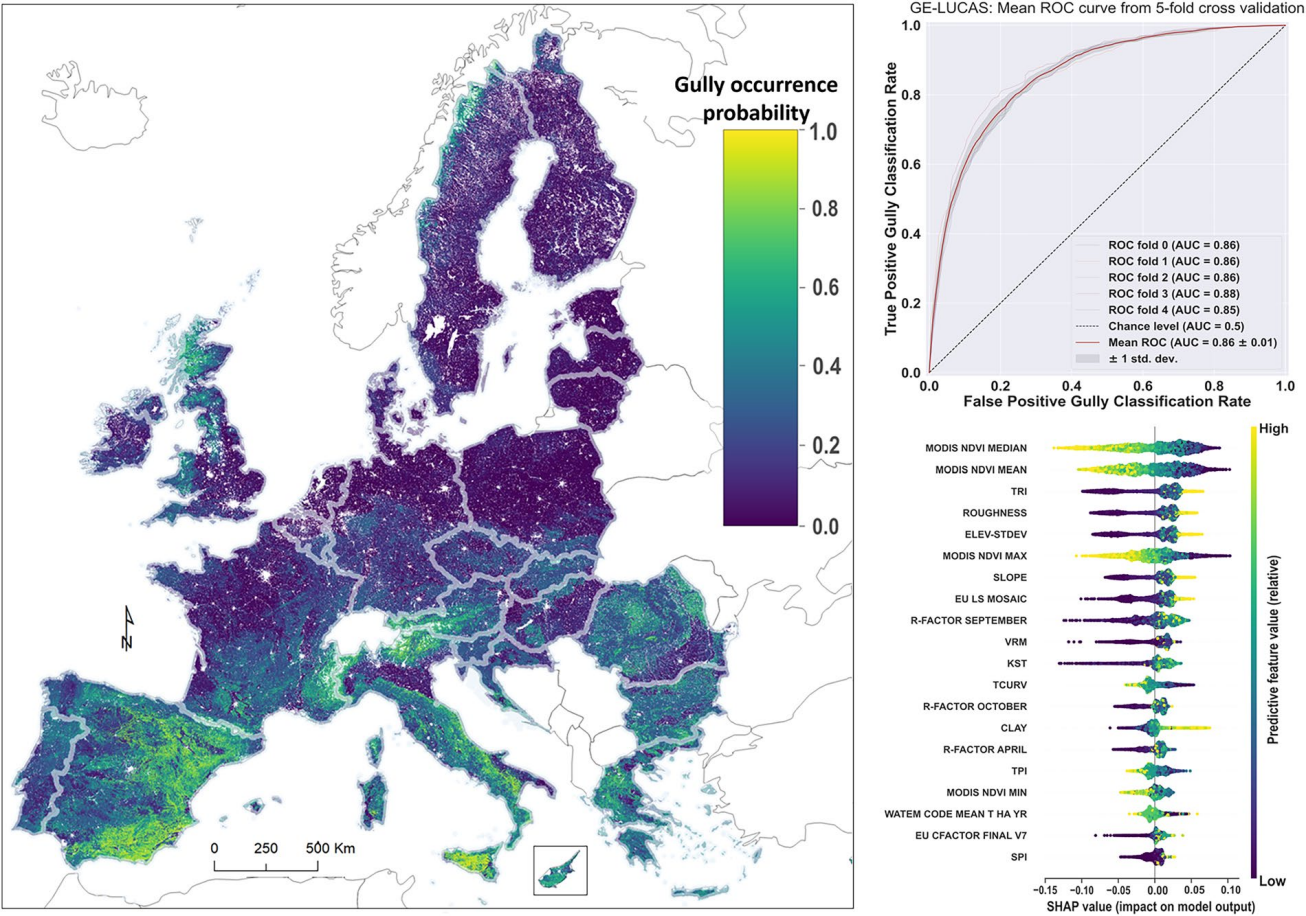
A summary of the: (**a**) the predictive interpolation of the gully erosion channels probability across the EU and the U.K. based on GE-LUCAS v1.1 points, (**b**) the individual and average Receiver Operating Characteristic (ROC) curves resulting from the stratified 5-fold cross-validation, and (**c**) a summary plot of the feature importance and their effects, according to the Shapley values. Features are ranked on the y-axis based on importance according to their absolute Shapley values, while individual Shapley value instances are plotted across the x-axis as a bee swarm plot.

We also preliminarily tested the potential of the GE-LUCAS v1.1 inventory to be used for estimating the probability of gully occurrence beyond the monitored locations. Figure 2 displays the probabilistic spatial interpolations of gully occurrence from a Random Forest (RF) classifier model in the EU and the United Kingdom (UK). Since no points from GE-LUCAS 1.1 cover the UK, these predictions represent an extra-domain extrapolation, laying the groundwork for future research that will reintegrate the UK while utilizing this preliminary map. Based on the validation portion of the dataset, the fitted classifier model had an accuracy of 0.78, an F1 score of 0.78, and an Area Under the Curve (AUC) of 0.87. A stratified 5-fold cross-validation furthermore rendered a mean AUC of 0.86. Feature importance, measured by the ranking of total Shapley values^15^, showed that vegetation properties (including median annual Normalised Difference Vegetation Index [NDVI], mean annual NDVI, and maximum annual NDVI), topographic properties (such as terrain ruggedness index, roughness, elevation standard deviation, slope, USLE slope length and steepness factors, and vertical ruggedness measure), soil properties (USLE soil erodibility factor with stoniness included), and rainfall dynamics (September USLE Rainfall Erosivity factor) were the most important features in the classifier model. The accompanying plot of Shapley instances furthermore shows the relative impact of each covariate on the model behaviour, allowing a visualisation of the relative forcing (positive or negative) of different feature values on the classifier model.

## Methods

### Data collection

**Fig. 3:**
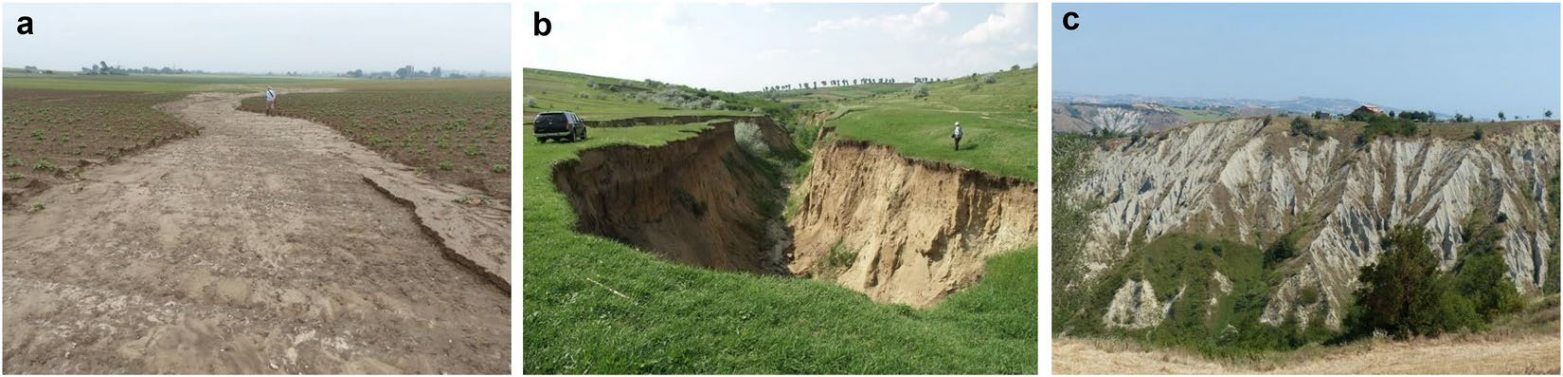
Examples of the types of gully erosion as shown in the LUCAS 2022 technical reference document. (**a**) Ephemeral gully formed in a bare potato field with ridges and furrows (near Neuville en Condroz, Belgium). (**b**) Permanent gully formed in a grassland valley bottom (near Barlad, Romania). (**c**) Badlands with multiple gullies forming a dense drainage network developed on Pleistocene clays (near Atri, Italy). All photos were provided by Jean Poesen.

LUCAS feature detection 2022 surveyed 399,591 locations through *in situ* (49.8%) and on-screen (50.2%) observations^1^. The gully erosion visual assessment of the LUCAS 2022 survey includes six of the 265 different variables collected: (i) presence or absence of gully channels; (ii) type of observed gully (ephemeral gully affecting only the topsoil, <0.5 m deep, permanent gullies affecting both topsoil and subsoil, 0.5–30 m deep, and Badlands (i.e., densely gullied landscape areas); (iii) direction of the gully from the LUCAS point (North, 315-45°, East, 45–135°, South, 135–225°, or West 225–315°); (iv) mean length of the gully in meters (<1, 1–10, 10–50, 50–100, 100–500); (v) mean width of the gully in meters (<1, 1–5, 5–10, 10–20, >20); and (vi) mean depth of the gully in meters (<0.3, 0.3–1, 1–5, 5–10, >10). In the LUCAS classification, the main differences between ephemeral and permanent gullies rest on the depth of the channel. The threshold for ephemeral gullies is set at a depth of 0.5 m, as these features are relatively easily obliterated by tillage operations. Badlands are understood as gully dominated landscapes that frequently form on poorly unconsolidated and low-permeability geological materials (like marls and clays). Figure 3 displays examples of three landforms with these corresponding gully types reported in the LUCAS 2022 technical reference document.

#### Spatial interpolation

The patterns of point-scale gully (non-)occurrence across the EU (Fig. 2) give informative but limited insights into their geographic occurrence. Given the low rate of gully presence within the LUCAS 2022 survey (0.8%), it can furthermore be argued that the lack of prior information on gully presence within the sampling design is poorly suited to identify topographically dependent gully features. Therefore, to provide spatially continuous insights into the probability of gully occurrence, a RF model was trained using the available GE-LUCAS v1.1 records to produce a continuous interpolation of the EU and the UK The RF implementation and evaluation was made within a standardised classification workflow in *Scikit-learn* (Pedregosa *et al*.^16^), and all code is publicly available. Firstly, the 3,116 points with gully presence were equally balanced by randomly down sampling the LUCAS-GE gully absence points, of which 50% of absence points were randomly sampled within the stratified climatic regions (EnS) with gully presence^17^, and a further 50% randomly sampled across the EU. Relevant spatial covariates were thereafter extracted covering a non-exhaustive but large diversity of relevant conditioning factors in the EU, taken from ESDAC^18^, geomorphometric layers at 90 m resolution^19^, and MODIS temporal composites processed in Google Earth Engine (TABLE AX). All layers were resampled to 100 m resolution prior to features extraction and model implementation. The decision to use a 100 m cell size resolution is based on the primary role of land use and land cover data (sourced from the 100 m CORINE dataset) and various topographical properties (derived from the EU-DEM with a 25 m resolution). In contrast, coarser resolution rasters (500–1000 m for rainfall and soil properties) have lower spatial relevance in the RF model.

Following covariate extraction at GE-LUCAS v1.1 points, a 70-30% train-test random split of the data was made to train and validate the RF classifier model. The RF predict probability (*predict_proba*) function was then applied to return the proportion of votes across the ensemble of trees for a given class, returning a probability between 0–1 of gully occurrence for each classified point. Hyperparameter tuning was undertaken on using the grid search to perform a randomised search over a parameter space for the best performing set of hyperparameters. Following hyperparameter tuning to the optimal values *(‘n_estimators’* = 7300, *‘min_samples_split’* = 10, *‘min_samples_leaf’* = 2, *‘max_features’* = ‘sqrt’, *‘max_depth’* = 80, *‘bootstrap’* = True), the RF model was implemented across the entire spatial domain at a 100-meter spatial resolution. Due to the relatively small data sample following class balancing, no tertiary test dataset was employed to evaluate the model predictions. Instead, a posterior mapping exercise was carried for 20 random locations per predicted probability class (0-0.2, 0.2–0.4, 0.4–0.6, 0.6–0.8, 0.8–1), each with a search radius of 1 km, to evaluate the validity of the predicted gully probability for informed mapping exercises.

## Data Records

The GE-LUCAS v1.1 data is available at Figshare^20^. In addition to Figshare^20^, the data and further information about LUCAS are also available through the European Commission’s Joint Research Centre, via the European Soil Data Centre (ESDAC): https://esdac.jrc.ec.europa.eu/content/gully-erosion-based-lucas. The data is stored in commonly used text and GIS file formats, such as comma-separated values (CSV), ESRI shapefiles, and GeoTIFFs. We georeferenced all files to the INSPIRE coordinate reference system, i.e., European Terrestrial Reference System 1989 (ETRS89-LAEA; EPSG:3035). We provide a catalogue in PDF format that includes an aerial image of each of the 3,116 observed gully erosion channels. The datasets have been organised as follows:

- **LUCAS2022_original.CSV** (411,845 KB) (comma-separated values format). This data format consists of 399,591 records that represents the locations of the LUCAS 2022 observations. The data is composed of 307 fields (columns) representing the 265 landscape elements included in the survey. Acronyms and data explanations are provided in the supporting file **LUCAS-2022-record-descriptor.ods** (22 KB).
- **LUCAS2022_with_gully_channels.shp** (2,998 KB) (shapefile format). This point geometry vector data model consists of 3,116 records representing the LUCAS2022 locations where gullies were detected. The vector data model includes the following fields: LUCAS code (POINT_ID), countries (POINT_NUTS), coordinates (POINT_LAT and POINT_LONG), altitude (POINT_ALTI) survey date (SURVEY_DAT), type of observation (SURVEY_OBS), land cover (SURVEY_LC1), land use (SURVEY_LU1), presence or absence of gullies (SURVEY_GUL), type of observed gully (SURVEY_G_1), direction of the gully from the LUCAS point (SURVEY_G_2), mean length in meters (SURVEY_G_3), mean width in meters (SURVEY_G_4), mean depth in meters (SURVEY_G_5). Further details are provided in Table 4.

**Table 4:** Description of the fields (variables) and attributes of the LUCAS2022_with_gully_channels and LUCAS2022_gully_channel_locations data.

Field Name	Options	Database field
LUCAS code	From 26381952 to 65041668	POINT_ID
EU country	27 European Union (EU) Member States	POINT_NUTS
Point latitude	Decimal degrees	POINT_LAT
Point longitude	Decimal degrees	POINT_LONG
Point altitude	Meters	POINT_ALTI
Survey date	Month/Day/Year	SURVEY_DAT
Type of Observation	1 Field survey, point visible, ≤100 m 2 Field survey, point visible, >100 m to point 3 Photo-interpretation in the field 4 Point not visible. PI not possible 5 Out of national territory 6 Out of EU 7 Photo-interpretation in the office	SURVEY_OBS
Land cover (LC1)	A: Artificial Land B: Cropland C: Woodland D: Shrubland E: Grassland F: Bare soil and Lichens G: Water Areas H: Wetlands	SURVEY_LC1
Land use (LU1)	U111 (agriculture): U112 Shrub areas in fallow land: U120 Forestry (Wood production): U140 (Small buildings used for mining and quarrying purposes): U210 (Small buildings used for energy production): U223 (Coal, oil and metal processing purposes): U224 (Production of non-metal mineral goods): U227 (Wood based products): U312 (Road transport on a viaduct): U313 (Bodies of water use for transport): U321 (Water supply and treatment): U361 (Greenhouses of botanical gardens): U370 (Individual residential houses): U411 (Abandoned industrial areas): U415 (Other abandoned areas): U314 (Air transport): U420 (Not used wooded areas)	SURVEY_LU1
Presence of gully	1 Yes 2 No	SURVEY_GUL
Type of gully	1 Ephemeral gully (<0.5 m deep) 2 Permanent gullies (0.5–30 m deep) 3 Badlands (i.e., gullied landscape areas)	SURVEY_G_1
Direction from point	1 North (315-45°) 2 East (45–135°) 3 South (135–225°) 4 West (225–215°)	SURVEY_G_2
Mean length in meters	(1, <1), (2, 1–10), (3, 10–50), (4, 50–100), (5, >100)	SURVEY_G_3
Mean width in meters	(1, <1), (2, 1–5), (3, 5–10), (4, 10–20), (5, >20)	SURVEY_G_4
Mean depth in meters	(1, <0.3), (2, 0.3–1), (3, 1–5), (4, 5–10), (5, >10)	SURVEY_G_5
- **LUCAS2022_gully_channel_locations.shp** (2,593 KB) (shapefile format). This point geometry vector data model consists of 3,116 locations found to be affected by gully erosion channels. The main difference with the ‘LUCAS2022_point_with_gullies’ vector data model lies in the fact that the points reported in this data do not reflect the LUCAS locations, but the locations where the actual gullies were found to be located. The vector data model includes the following fields: LUCAS code (POINT_ID), countries (POINT_NUTS), survey date (SURVEY_DAT), type of observation (SURVEY_OBS), land cover (SURVEY_LC1), type of observed gully (SURVEY_G_1), mean width in meters (SURVEY_G_4), and mean depth in meters (SURVEY_G_5). For details refer to Table 4.
- **Europe_RF_gully_probability_map.tif** (597,201 KB) (GeoTIFF format). This data represent the probabilistic spatial interpolations of gully occurrence from the RF classifier model in the EU and the UK. The map is provided as a percent probability layer in integer format.
- **Random_cross-check.shp** (237 KB) (shapefile format). This point geometry vector data model consists of 4,500 randomly selected locations (~1% of the LUCAS 2022 records) for which the presence or absence of gullies has been determined via a further on-screen visual assessment of Google Earth™ imagery. There is only one field (i.e., Code) in this vector data model, and its values can be 0 (no gully) or 1 (yes gully).
- **LUCAS2022_cross-check.shp** (106 KB) (shapefile format). This point geometry vector data model comprises of 2,500 LUCAS 2022’s locations revisited through on-screen visual assessment of Google Earth™ imagery to gain statistical indication of the error of omission (potential false negatives).
- **GE-LUCAS v1.1 aerial photo inventory.PDF** (47,293 KB) (text format). A library of aerial photos of all the 3,116 LUCAS 2022 locations in which gullies were observed in the *in situ* or remote observations (Fig. 4).

**Fig. 4:**
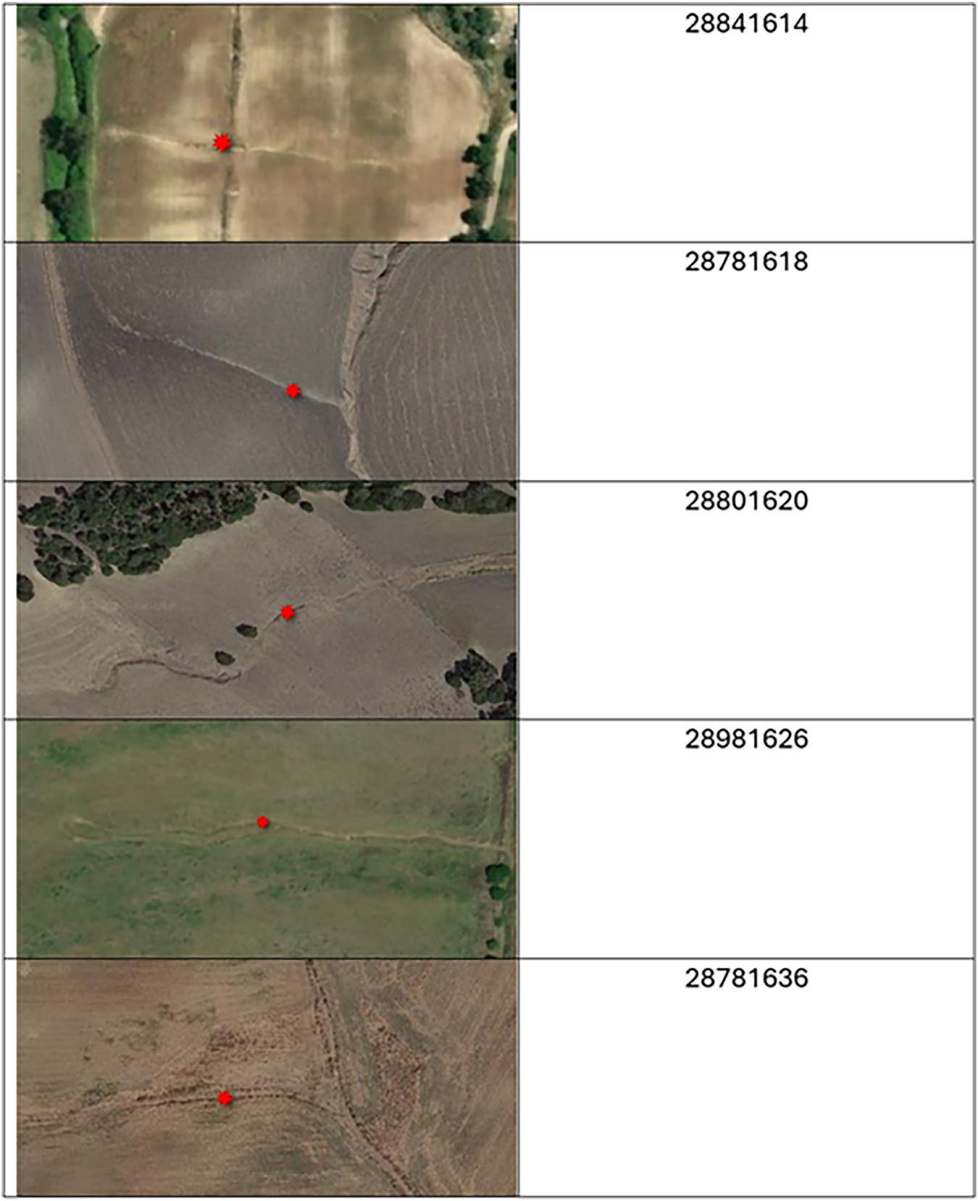
GE-LUCAS v1.1 aerial photo inventory. Th show five landscapes in Spain of the 3,116 LUCAS 2022 locations where at least one gully channel was detected via in situ and on-screen surveys. Numbers on the right represent the LUAS ID of each location. Background imagery: Google Earth™.
- **LUCAS ground photo archive** (5,670,000 KB) (JPG format). The ground photo archive includes 8,488 out of approximately 15,580 total LUCAS cover photos (five per LUCAS location). The discrepancy between the number of photos collected and the total is due to the unavailability of some photos in the Eurostat repository. This may be attributable to privacy concerns or other formal constraints.

## Technical Validation

**Fig. 5:**
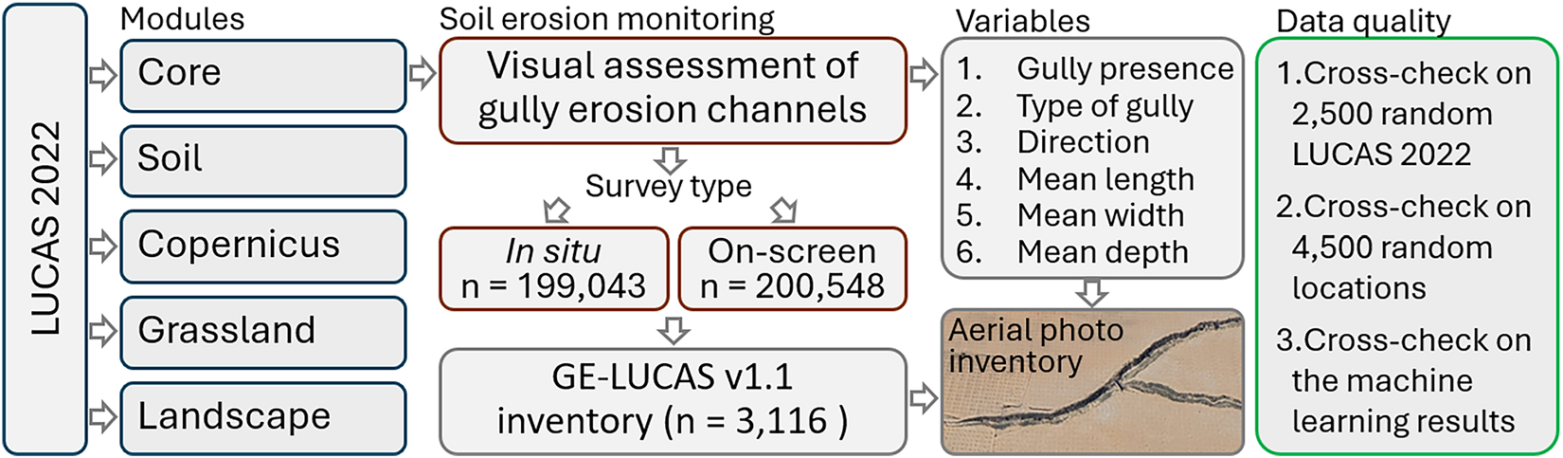
An overview of the soil erosion monitoring procedure included in the LUCAS 2022, including its main processing steps and outcomes.

Figure 5 provides a summary of the LUCAS 2022 survey, the acquired variables regarding gully erosion channels, and the used data quality control method to assess the potential accuracy of gully mapping and spatialization.

### Cross-checking

**Fig. 6:**
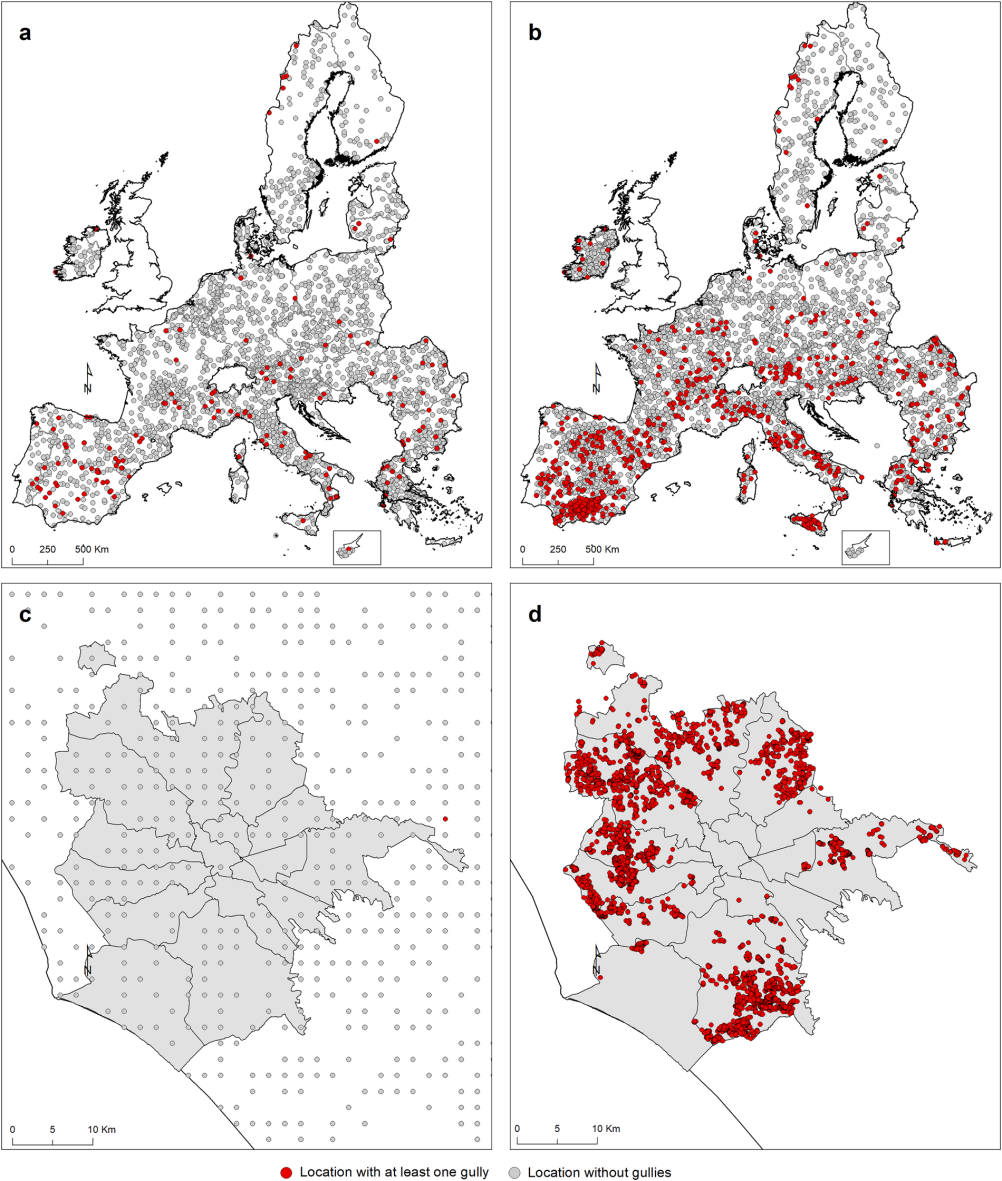
Datasets used for verifying the accuracy and consistency of the LUCAS 2022 survey data. (**a**) The 2,500 randomly selected LUCAS 2022 locations used to assess the error of omission (possible false negatives). (**b**) The 4,500 randomly selected locations (excluding LUCAS) used to validate the spatial patterns and consistency of the LUCAS 2022 survey. (**c**) LUCAS 2022 locations situated around the Municipality of Rome (Central Italy). (**d**) Gully features (i.e., gully heads) mapped through on-screen visual detection of the entire arable land area of the Municipality of Rome (2018–2024).

We conducted a cross-check on 2,500 randomly selected LUCAS 2022 locations where the surveyors did not report any gully erosion channels. To accomplish this, we utilised Google Earth™ imagery. The goal of this validation procedure was to statistically assess the error of omission, which refers to possible false negatives occurred during the survey. A square observation area measuring 300 by 300 m was established around each location. The size of the observation area was defined based on the LUCAS straight line transects (250 m) and the typically reported gully distances from the LUCAS observation point (≤100 m). When at least one gully channel was recorded through a thorough expert-based remote verification (using high-resolution aerial images), the location was classified as a possible false negative. According to the on-screen visual assessment, 139 out of the 2,500 revisited LUCAS 2022 locations (or 5.6% of the total) showed at least one gully erosion channel (Fig. 6a). A figure consistent with the omission error observed in the LUCAS 2018 topsoil survey^3^.

We conducted a further cross-check on 4,500 randomly selected locations (~1% of the LUCAS 2022 locations). Also in this case, gully erosion channel detection was performed through on-screen visual interpretation of the most recent Google Earth™ imagery. During the selection of random locations, a minimum distance of 1,000 m from the LUCAS 2022 locations was defined. The purpose of this additional validation was to verify that the ability to detect gully erosion channels of the LUCAS surveyors is comparable to that of a trained geomorphologist. Also in this case, we established a square observation area measuring 300 by 300 m around each site. We recorded 605 locations with gully erosion channels (Fig. 6b), which is equal to an occurrence rate of ~13.5%. In general, the geographical gully erosion patterns resulting from the cross-check analysis aligns well with the findings of the LUCAS study. One element that arises from this analysis is the potential underestimating of gully erosion channels in the LUCAS 2022 survey, which seems substantiated by the higher gully occurrence rate of gullies (~13.5%) combined with the observed omission error (5.6%) observed in these validation exercises. A hypothesis supported when comparing the results of the LUCAS 2022 survey (Fig. 6c) with a high temporal frequency (2018–2024) on-screen visual detection (Fig. 6d) conducted for the municipality of Rome (Italy). Although the LUCAS 2022 survey does not report any gully erosion channels in Rome, our extensive mapping efforts (covering the entire arable land (approximately 400 km^2^)) identified a total of 6,354 gully features (i.e., gully heads).

### Interpolation

The potential of the pan-EUinterpolation to identify areas with varying levels of gully occurrence was tested via a posterior mapping exercise. Firstly, 100 locations with a search radius of 1 km were randomly selected based on their mean probability class, split into 20 locations for each of 5 different probability classes. Thereafter, a mapping exercise was undertaken based on the available high resolution satellite imagery in Google Earth^TM^ to count the total number of visible gully heads per location. This exercise was intended to evaluate the potential of machine learning predictions based on the already available GE-LUCAS v1.1 data resource to inform future ground and/or remotely based mapping exercises targeting gully presence. Importantly, based on the current prediction accuracies combined with potential commission and omission errors within GE-LUCAS v1.1, a high or low gully probability does not guarantee the presence or absence of gully erosion features. However, these give probabilistic insights into the areas of the EU and the UK in which gully presence is relatively more likely based on the current GE-LUCAS v1.1 resource.

**Fig. 7:**
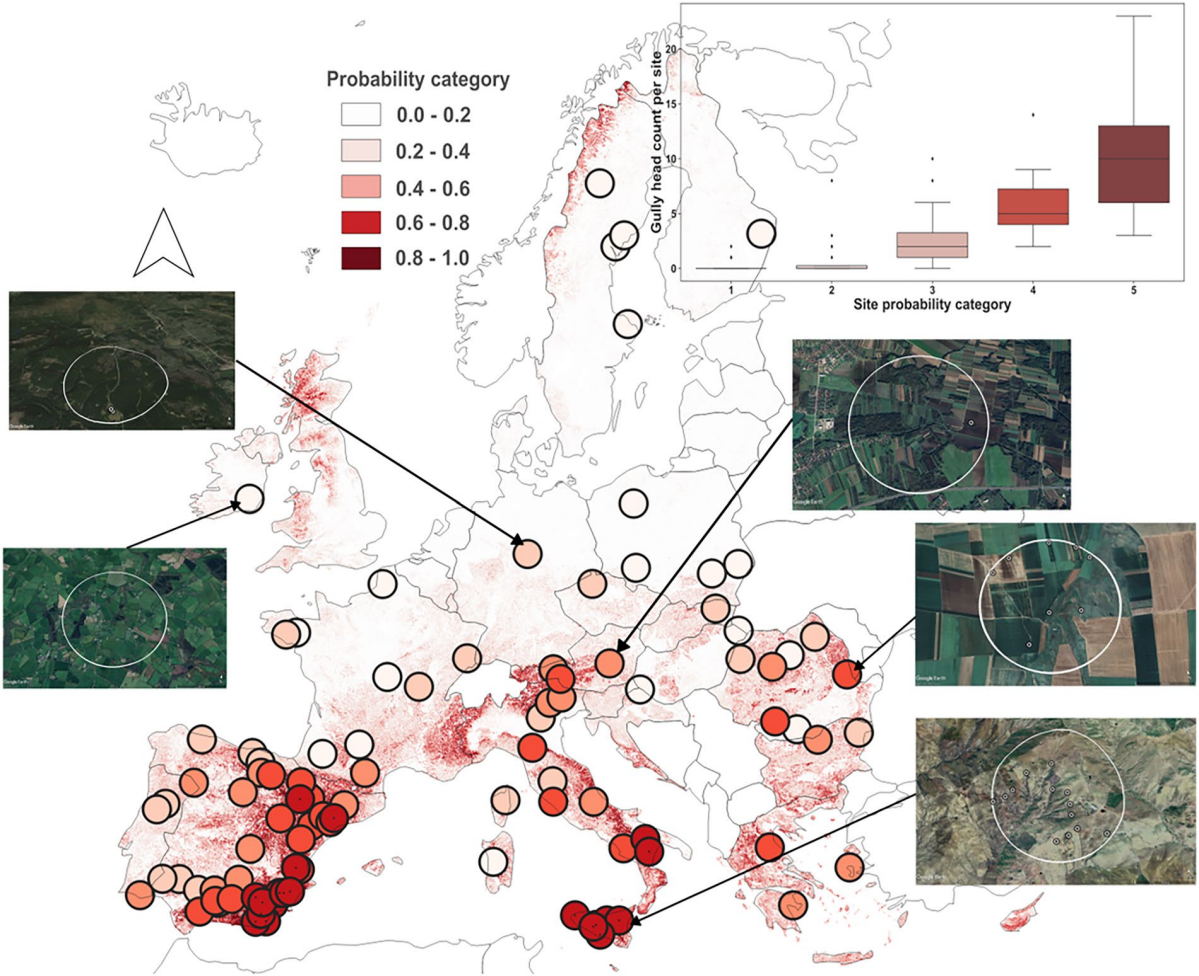
An overview of the secondary validation exercise to assess the potential of the interpolated estimates of gully occurrence probability to locate gully-prone areas in the EU. Coloured points depict the 100 sites with a 1 km radius which were randomly selected according to their mean probability of gully occurrence. The accompanying boxplot (upper right) summarises the distribution of counted gully heads per site per probability category. Examples of mapped locations are given for each probability class using image exports from Google Earth^TM^.

Figure 7 evidences the utility of the probability estimates from spatial classifiers to provide indicative predictions, identifying areas with gully occurrence based on a non-exhaustive set of observations. A clear difference in the count of mapped gully features per location can be observed between the different probability categories, inferring that these predictions can guide more extensive mapping efforts in the future. The current spatial predictions have several potential uses as a data resource to: (i) derive acceptably accurate indications of gully presence/absence based on the current GE-LUCAS v1.1 data record, (ii) target new gully presence locations outside of LUCAS survey locations, which may support future data-driven model efforts by reinforcing the available data resource, and (iii) a resource to guide cross validation exercises of locations with a surveyed gully absence in LUCAS.

### Limitations of the survey and spatial interpolation

The importance of data resources in data-driven modelling means that future reinforcement of the GE-LUCAS v1.1 gully inventory resources may provide opportunity to address unresolved but pertinent issues for pan-EU gully erosion modelling. These include, but are not limited to: (i) the issue of under-represented ephemeral gullies in GE-LUCAS v1.1, which currently limits the resource for spatiotemporal modelling exercises and likely introduces spatial bias within the gully presence observations^21^ (ii) the lack of data on active retreat rates, limiting possible predictions of gully expansion and erosion rates, (iii) tackling presence-absence class imbalance issues in naturally class-imbalanced classification problems such as gully erosion^22,23^, for example the exploration of oversampling, class weighting, and varied cutoff probability threshold techniques on the spatial predictions, (iv) issues obtaining high probability absence data for supervised learning models when observations are made in a limited temporal window, limiting the theoretical assignment of a true probability of 0 to some absence points^24^. Further developments of the GE-LUCAS v1.1 resource will support opportunities to apply model interpretability techniques to generate hypotheses to derive new understanding from data-driven models applied across the EU (Jiang *et al*.^25^). Overlapping with many of the aforementioned focal points, future modelling tasks may further benefit in both accuracy and spatial representativeness from using spatiotemporal covariates at a higher resolution compared to this current application (100 m).

GE-LUCAS v1.1, based on LUCAS 2022 survey, represents a significant advancement in understanding and detecting gully erosion patterns across Europe. However, several key elements for improvement have emerged that would further enhance the monitoring capacity of gully erosion in the future: (i) a single snapshot sample design is insufficiently adapted to detect ephemeral gully features which develop post-rainstorm and are easily removed by tillage or quickly covered by vegetation growth, (ii) the absence of active retreat rates restricts the prediction of gully expansion and erosion rates, and finally (iii) the imbalance in classes’ occurrence, which is a longstanding challenge in classification problems. On a similar note, the timing of LUCAS surveying (starting in the South of Europe in spring and finishing in the North later in the year) is generally scarcely aligned to the occurrence of ephemeral gullies which have higher probabilities in certain seasons for different parts of Europe. A condition that may support the lack of gully evidence observed around Rome in the LUCAS2022 survey. Finally, we believe that high resolution satellite imagery may be more suited to the task of locating gully presence, especially if aided by prior spatial probability estimates used to build a stratified random sampling. A monitoring strategy that, in light of our findings, may result in more efficient monitoring of Europe’s gully erosion channels compared to the regular fishnet network adopted in LUCAS 2022. Based on the insights gained, we propose that the next phase of the pan-European Union assessment of gully erosion channels should involve a stratified random monitoring approach, conducted on-screen, across an additional 100,000 to 200,000 locations. The strata can be defined according to the predictive factors identified by the RF analysis. Moreover, these on-screen evaluations should be conducted more frequently, allowing for better detection of the dynamics of gully occurrence over time.

## Data Availability

The inventory of gully erosion channels was created and verified without the need for any custom coding. A publicly accessible repository contains the custom code that was used to create the gully occurrence probability map (Fig. 2) and the ground photos from the LUCAS archive (GitHub^26^). Codes for producing the gully occurrence probability map were written using Spyder 6.0 free and open-source software.
